# Integrating AI and genomics: predictive CNN models for schizophrenia phenotypes

**DOI:** 10.1515/jib-2024-0057

**Published:** 2025-06-18

**Authors:** Guilherme Henriques, Maryam Abbasi, Daniel Martins, Joel P. Arrais

**Affiliations:** Department of Informatics Engineering, University of Coimbra, CISUC/AC – Centre for Informatics and Systems of the University of Coimbra, Coimbra, Portugal; Polytechnic Institute of Coimbra, Coimbra, Portugal; Research Centre for Natural Resources Environment and Society, Polytechnic Institute of Coimbra, Coimbra, Portugal; Biocant – Transfer Technology Association, Cantanhede, Portugal

**Keywords:** schizophrenia, deep learning, phenotype prediction, Convolutional Neural Networks, CNN

## Abstract

This study explores the use of deep learning to analyze genetic data and predict phenotypic traits associated with schizophrenia, a complex psychiatric disorder with a strong hereditary component yet incomplete genetic characterization. We applied Convolutional Neural Networks models to a large-scale case-control exome sequencing dataset from the Swedish population to identify genetic patterns linked to schizophrenia. To enhance model performance and reduce overfitting, we employed advanced optimization techniques, including dropout layers, learning rate scheduling, batch normalization, and early stopping. Following systematic refinements in data preprocessing, model architecture, and hyperparameter tuning, the final model achieved an accuracy of 80 %. These results demonstrate the potential of deep learning approaches to uncover intricate genotype-phenotype relationships and support their future integration into precision medicine and genetic diagnostics for psychiatric disorders such as schizophrenia.

## 1 Introduction

Schizophrenia (SCZ) is a complex and multifaceted psychiatric disorder that impacts millions globally, leading to substantial disability and imposing a heavy strain on affected individuals, their families, and healthcare system [1], 2]. It is characterized by disruptions in cognition, emotion, and perception, and its multifactorial nature – stemming from genetic, environmental, and physiological factors – poses significant challenges for effective diagnosis and treatment [3]. To address these challenges, a robust approach combining genomics, epidemiology, bioinformatics, and clinical research is necessary [4]. Research into the genetic basis of SCZ has shown a vital hereditary component, with family and twin studies estimating a heritability rate of approximately 80 % [5], 6]. Although considerable research has been conducted, the precise genetic contributors to SCZ remain elusive. Numerous genes, particularly those regulating neurotransmitter pathways – such as dopamine, glutamate, and GABA – have been implicated as potential susceptibility factors. Nonetheless, their exact contributions to the onset and progression of SCZ require further elucidation [7], [8], [9], [10]. This persistent ambiguity underscores the intricate nature of the disorder and necessitates the adoption of more sophisticated methodologies to dissect its genetic foundations. SCZ is clinically diagnosed primarily through the assessment of characteristic symptoms, including hallucinations, delusions, cognitive deficits, and social disengagement. However, these manifestations exhibit substantial variability across individuals and frequently overlap with symptoms of other psychiatric conditions, complicating the diagnostic process [11]. Misdiagnosis is not uncommon, and this not only delays appropriate treatment but also complicates efforts to elucidate the genetic mechanisms underlying SCZ [12], 13]. In response, several large-scale case-control studies, particularly those conducted in Scandinavian countries, have sought to better understand the biological aspects of SCZ by focusing on its genetic risk factors [14], 15].

In the past decade, machine learning (ML) has gained traction as a powerful approach for analyzing and interpreting complex biological data, including genetic data [16], [17], [18]. ML models have the ability to detect intricate patterns in large genomic datasets that might be overlooked by traditional statistical methods [19], 20]. This capability makes ML particularly well-suited for studying complex diseases like SCZ, where multiple interacting genetic and environmental factors are at play [21], 22].

The application of ML in psychiatry has shown great potential in helping the early diagnosis of SCZ. By analyzing genetic profiles, environmental exposures, and clinical history, ML algorithms can identify individuals at high risk for developing SCZ [23], 24]. These predictive models hold the potential to revolutionize clinical practice by facilitating earlier and more precise diagnoses. This advancement paves the way for personalized treatment approaches tailored to an individual’s specific risk profile [25], 26].

The main goal of this research is to develop a computational model that utilizes ML algorithms to identify genetic patterns linked to SCZ just based on genetic data [25], 26]. By optimizing data preprocessing techniques and leveraging existing biological knowledge, we seek to improve the accuracy and interpretability of our models, thus contributing to a deeper understanding of the genetic mechanisms underlying SCZ. The Python code used in this study is available at https://github.com/larngroup/CNNSCZ.

## 2 Methods

### 2.1 Convolutional Neural Network (CNN) model architecture

This study utilizes a Convolutional Neural Network Architecture, a deep learning model tailored for processing data with grid-like topology, commonly employed for image and pattern recognition tasks [27]. CNNs are highly efficient for feature extraction due to their hierarchical structure, low number of trainable parameters compared to fully connected networks, and adaptability in learning abstract data representations [28]. This architecture has been expanded to applications beyond images, including genomic sequence data, which exhibits a regular grid structure, making CNNs well-suited for this task [29].

**Figure 1:**
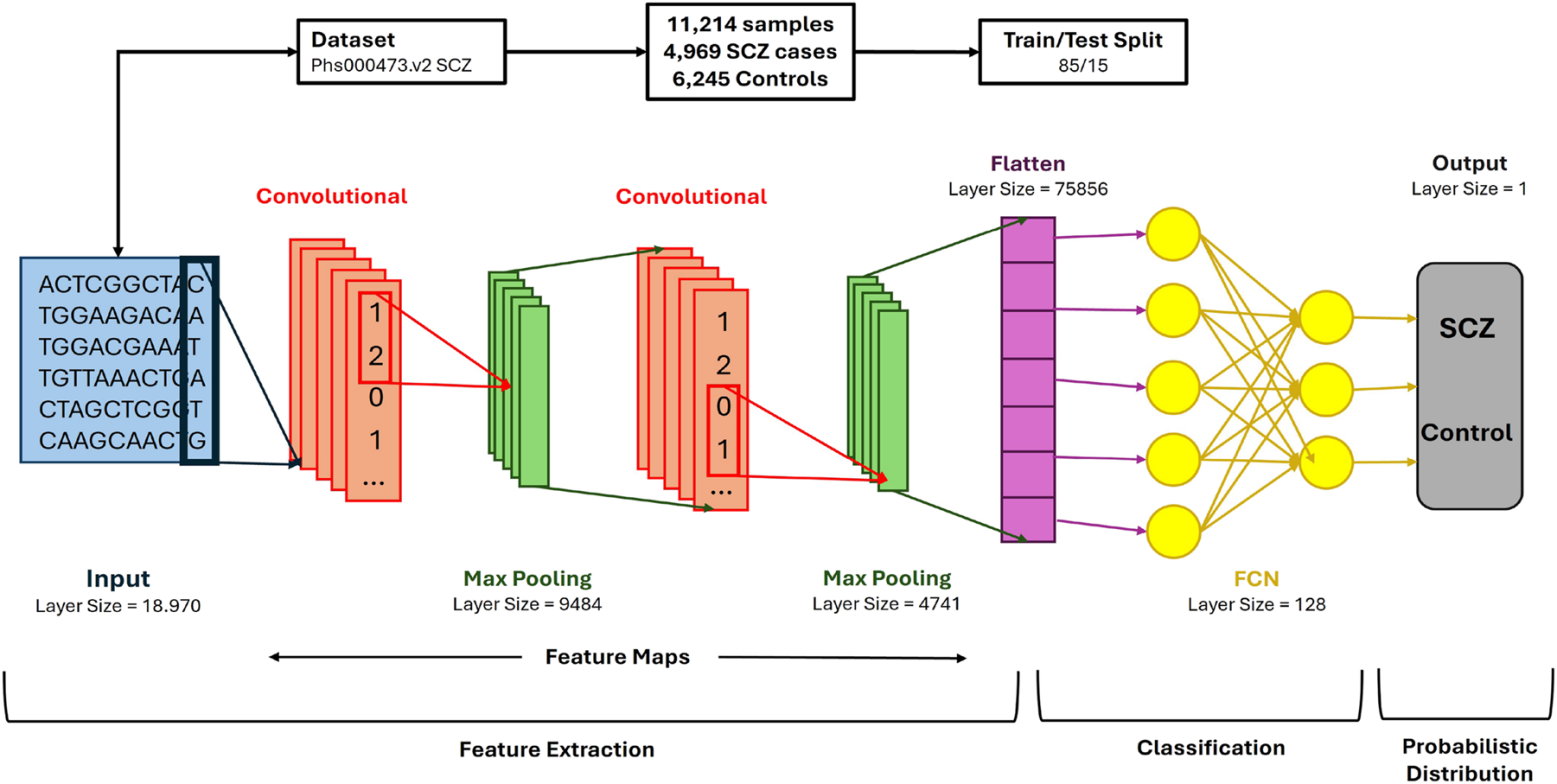
Convolutional Neural Network Structure: the designed architecture efficiently extracts patterns in genomic data to predict outcomes based on genome variants of each individual.

The proposed model is structured with multiple layers, each playing a crucial role in identifying complex patterns within genomic data. The model architecture comprises two convolutional layers, each seamlessly integrated with a max-pooling layer to reduce dimensionality and minimize overfitting effectively. This configuration enables the model to capture intricate and broader genomic patterns while preserving a compact and efficient design. As shown in Figure 1, the CNN starts with a 2-dimensional (2D) convolutional layer, ideal for handling the structured input data despite the dataset’s one-dimensional nature since 2D convolutions allow for better spatial feature extraction. A 2D convolutional layer was chosen to capture the spatial patterns in the genomic data, even though our dataset is one-dimensional, meaning that the DNA sequences are represented as linear strings. The 2D approach is ideal for this case because, in the 1D one, the model would only be able to scan along the length of the sequence, which would limit the model’s ability to detect the interactions between more distant regions; however, by using a 2D convolution, we can reshape the 1D data by organizing the genomic sequences into smaller segments, allowing the model to learn patterns across both the sequence and different features (segments) simultaneously, ending up learning both local dependencies and higher order spatial relationships [30].

The addition of more than one convolutional layer allows the network to learn a hierarchical feature representation. Early layers focus on understanding simple patterns, such as edges or basic genomic motifs, while deeper layers capture more complex features, such as interactions between genetic variants [31]. To further mitigate underfitting, additional convolutional layers were incorporated, thus increasing the feature learnability by deepening the network structure [32].

Max-pooling layers, placed after each convolutional layer, are crucial in reducing the spatial dimensions of the feature maps, which aids in controlling overfitting by summarizing regions of the input [33]. These pooling layers enhance the network’s ability to generalize by down-sampling the feature maps, making the network less sensitive to minor variations in the input data, a typical issue in biological datasets [34].

Finally, after the convolution and pooling layers, a fully connected (FC) layer is included to aggregate the extracted features and pass them to the output layer for classification [35]. The FC layer transforms the multidimensional feature maps into a one-dimensional vector, which is subsequently used for classification tasks. The output layer uses a softmax activation function to predict the class probabilities, such as the presence or absence of a genetic condition, based on the input genome variants [36].

Once the architecture is implemented, the model undergoes training using a labeled dataset, and its performance is evaluated using metrics such as accuracy and loss. These metrics are indicative of the model’s predictive capability and efficiency in classifying genomic data [37]. Further refinements, such as hyperparameter tuning and regularization, are applied to optimize the network’s performance during training [38].

### 2.2 Dataset and preprocessing

In this study, we utilized data from the *Sweden Schizophrenia Population-Based Case-Control Exome Sequencing* dataset, which is stored in the dbGaP repository [39] under the accession ID code phs000473.v2.p2. The dataset includes information for 12,380 individuals, consisting of 6,245 controls and 6,135 cases. Of the cases, 4,969 were diagnosed with SCZ, while 1,166 were diagnosed with bipolar disorder. However, for the purposes of this study, only the SCZ cases were analyzed to maintain a binary classification framework. Participants in both the case and control groups were required to be at least 18 years old, with both parents born in Scandinavia [40], [41], [42]. Participants for the study were selected through data from the Swedish Hospital Discharge Register. To qualify, individuals need to have been hospitalized on at least two separate occasions, with each admission confirming a diagnosis of schizophrenia (SCZ). Any individuals diagnosed with other medical or psychiatric disorders that might compromise the precision or reliability of the SCZ diagnosis were not included in the research sample. Control cases were randomly selected from the general population and excluded if they had any recorded history of hospitalization for SCZ. To the best of our knowledge, this dataset represents one of the most considerable whole-exome and whole-genome sequencing resources available for studying schizophrenia, accessed through request.

The initial dataset comprised 1,811,204 variants, all of which had a call Phred-score (*QUAL*) above 30 [43]. For this study, BCFtools [44], along with its *fill-tags* plugin, was utilized to remove the bipolar samples from the dataset and to recalculate key metrics for each variant site, including mean read depth (*DP*), Allele count (*AC*), Number (*AN*), and frequency (*AF*). Further filtering was carried out using VCFTools (Version 0.1.15) [45], applying criteria such as allele count (*AC* = 0), depth (*DP* < 8), and a genotype call rate lower than 90 %. Multi-allelic variants were segregated with BCF tools. For newly identified variants, genotypes that were neither the reference nor the alternative allele were marked as missing. A second round of filtering was applied to the split variants, adhering to the same conditions: allele count (*AC* = 0), depth (*DP* < 8), and genotype call rate under 90 %. The Variant Quality Score Recalibration process from the Genome Analysis Toolkit Best Practices Workflow (Version 3.8) [46] was followed to guarantee variant call accuracy.

In order to explore potential relationships between genotypic variation and phenotypic expression, chi-squared tests were performed utilizing a 3 × 3 contingency framework, accommodating the three possible genotypic categories across both case and control groups. Variants located on sex chromosomes, as well as insertion-deletion polymorphisms (InDels), were deliberately excluded to maintain consistency and reliability in the statistical assessment. The resulting *p*-values were reported without correction for multiple testing. This decision was made to preserve a broader spectrum of genetic variants for subsequent evaluation, reducing the likelihood of overlooking variants that may exert meaningful biological influence. By adopting this more inclusive strategy, the analysis allowed for a more exhaustive examination of potential genotype-phenotype associations. After filtering, the dataset contained 18,970 variants, which were then annotated using the most up-to-date version of Annovar [47], aligned to the hg19 genome RefSeq [48], as of October 19, 2021. The annotated variants spanned 9,160 unique genes.

## 3 Results and model optimization

The model development involved multiple trial and error stages, spanning approximately 43 h (2,550 min) of testing. After each test, the results were analyzed to identify potential issues or factors affecting the model’s performance. The initial code was more straightforward and less structured, but the model’s precision improved significantly with subsequent parameter tuning and the addition of layers and techniques. Visual representations, such as graphs, were used to illustrate the model’s output and performance effectively. The results are explained below based on the different stages of model refinement.

### 3.1 Initial model testing

In the first version, we allocated 99 % of the data for training and only 1 % for testing. While this model achieved an accuracy of 100 % with an impressively low loss value of 4.9557 × 10^−5^ %, these results were achieved within 6–9 training epochs. This raised concerns about overfitting, as such rapid convergence often indicates that the model may not generalize well to new data. To counter this, we explored parameter adjustments and model architecture modifications, aiming to improve its ability to generalize effectively.

### 3.2 Optimized data split and architectural refinements

Through a series of adjustments, we achieved more reliable results. We modified the data split to allocate 85 % for training and 15 % for testing, reduced the layer sizes, and increased the number of epochs from 15 to 20. Additionally, we expanded the number of neurons in the fully connected network (FCN) from 100 to 500. To improve clarity, we also simplified the way predictions were displayed, making performance analysis more intuitive. These refinements resulted in a more steady accuracy progression over epochs, with the loss value stabilizing at approximately 2 %. However, despite these improvements, around 50 % of the predictions remained incorrect, highlighting the need for further optimization.

### 3.3 Parameter optimization and additional refinements

To further enhance the model, we increased the number of epochs to 100 and introduced a new parameter: the percentage of data used for predictions. Additionally, we added an extra neuron layer to the FCN, incorporated dropout in both the max-pooling layers and the FCN, and implemented a function for graphical result visualization. These modifications led to more stable training performance, with accuracy and loss following expected trends. The testing accuracy improved to 68 %, while the prediction error rate decreased to 10 %. However, despite these enhancements, graphical analysis of the testing set still suggested the presence of overfitting, albeit at a reduced level.

### 3.4 Overfitting reduction and model optimization

To further minimize overfitting, we simplified the FCN by removing its second neuron layer and introduced an optimization technique. We tested both the “adam” and “SGD” optimizers and ultimately chose “adam” since the performance difference between the two was negligible. The modifications led to noticeable improvements in the testing set graphs, with smoother accuracy and loss curves showing fewer fluctuations. Accuracy saw a slight increase of approximately 1 %, while the prediction error dropped from 10 % to 8 %. Additionally, incorporating early stopping allowed the model to assess performance at each epoch and discard those that did not contribute to accuracy gains. This adjustment further stabilized the loss and accuracy curves, leading to more consistent and reliable performance.

### 3.5 K-Fold cross-validation and model adjustments

Following extensive testing, we introduced modifications to the model by incorporating a third dimension of size three, where each layer corresponded to a specific genotype (0, 1, and 2) [49]. Instead of using pre-trained weights, genotype-specific values were assigned, while the remaining layers were initialized with zeros. These adjustments produced accuracy and loss values comparable to previous tests, with slight improvements in learning curves. However, overfitting persisted, prompting the integration of K-fold cross-validation [50].

Even with the implementation of K-fold cross-validation, graphical analyses continued to indicate the presence of overfitting. Since the modifications did not make substantial improvements, we began exploring alternative approaches to enhance model generalization.

### 3.6 Final model

To further enhance performance, we experimented with various regularization methods, including Drop Blocks [51] and Spatial Dropouts. However, these approaches had minimal impact on the model’s performance. We also tested an attention mechanism [52] to improve both accuracy and interpretability, but the results did not show significant improvements. Ultimately, we opted to integrate Batch Normalization across most layers while maintaining dropout rates at 0.5.

One of the key breakthroughs came from implementing a Learning Rate Schedule, which dynamically adjusted the learning rate between epochs. This strategy helped address the early plateau observed with early stopping, leading to a notable improvement. As a result, model accuracy increased to 76 %, and the learning curves demonstrated better convergence, indicating enhanced stability during training.

**Figure 2:**
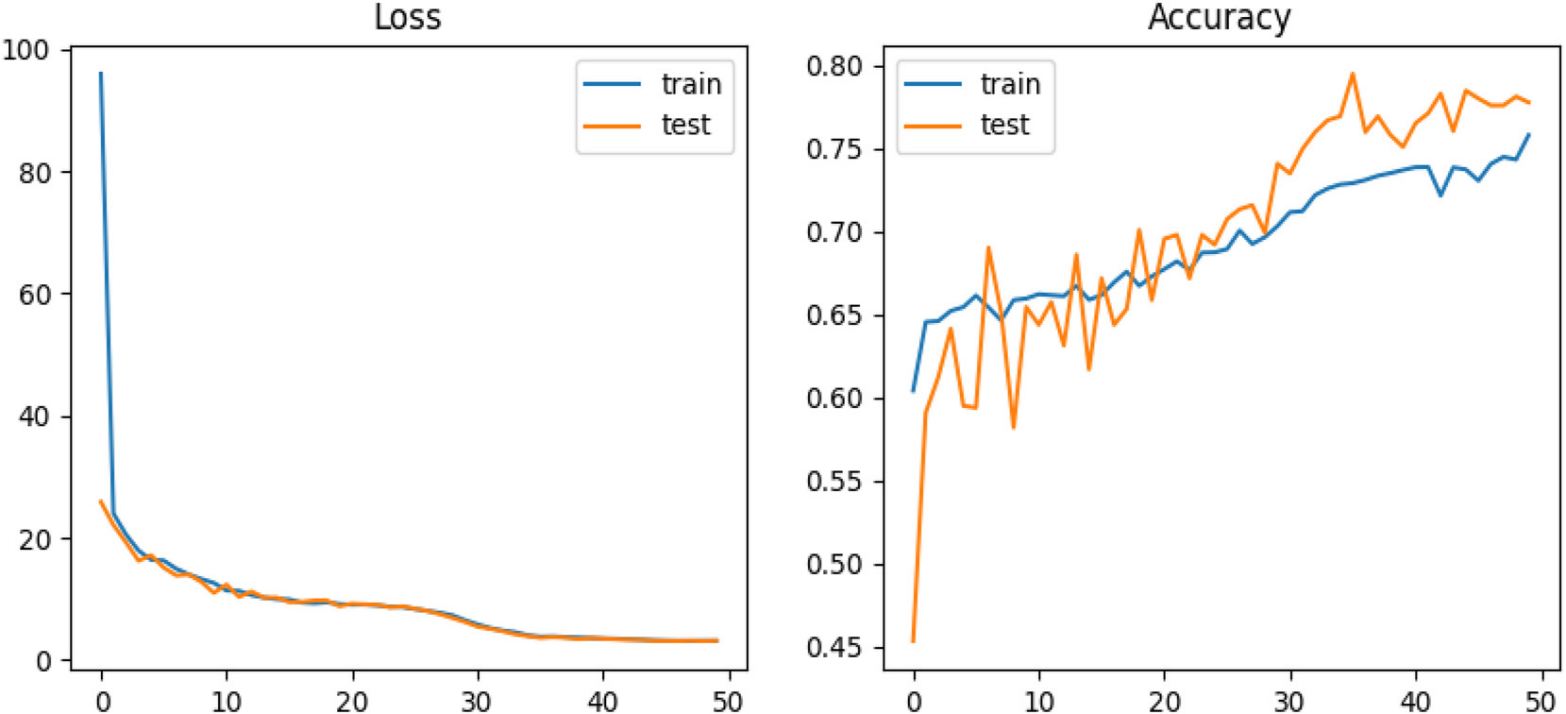
Performance evaluation of the final 2D model over 50 epochs, incorporating a single neuron layer with 1,000 neurons, a learning rate schedule, and a learning rate reduction function. The figure illustrates model accuracy, prediction error percentage, and learning curves for both training and testing sets.

In the final phase of model development, we introduced a Learning Rate Reduction function that dynamically decreased the learning rate when progress stagnated. To simplify the architecture, we used a single neuron layer with 1,000 neurons and reduced the number of filters. These refinements led to the highest recorded accuracy of 80 % (see Figure 2).

The learning curves for both the training and testing sets showed significant improvements, reflecting a more balanced ratio of correct to incorrect predictions and a noticeable reduction in overfitting. To evaluate the impact of different model configurations on performance, we tested several combinations of model dimension, filter sizes, and batch sizes. The results, presented in Table 1, highlight the most effective setups for both 2D and 3D CNNs. Additionally, we investigated the influence of varying neuron counts in different layers across both architectures. As shown in Table 2, increasing the number of neurons yielded only marginal improvements in accuracy, with no significant gains beyond a certain threshold.

**Table 1:** Analysis of different model configurations, including variations in model dimension (MD), filter sizes, and batch sizes, and their impact on performance.

ID	MD	Filters	Batch size	Train Acc.	Test Acc.	Observations
1	2D	64	64	99.56	72.06	Baseline reference
2	2D	32	32	96.93	70.39	Best parameter combination
3	2D	64	32	99.9	72.29	Highest accuracy for both sets
4	2D	32	64	99.51	71.28	Second-best results
5	2D	64	32	86.28	69.08	Reference for ID – 3
6	2D	64	32	99.39	64.1	K-fold validation applied to ID – 3
1	3D	64	64	91.56	70.89	Highest test accuracy in 3D
2	3D	64	64	98.42	69.42	Best train-test accuracy balance
3	3D	32	32	90.45	70.22	Lowest overall accuracy
4	3D	64	32	90.8	70.49	3D model reference: ID – 2
5	3D	32	64	92.78	70.44	K-fold applied to 3D model: ID – 2

**Table 2:** Effect of varying neuron counts in different layers for 2D and 3D CNN structures. MD: Model Dimension, N1: Neurons in layer 1, N2: Neurons in layer 2, Tr. Acc.: Train Accuracy, Ts. Acc.: Test Accuracy, Obs.: Observations.

ID	MD	N1	N2	Tr. Acc.	Ts. Acc.	Obs.
1	2D	500	100	98.76	70.34	Minimal impact on accuracy
2	2D	1,000	500	99.12	71.56	Slight improvement observed
3	2D	1,000	1,000	99.34	71.89	No significant accuracy gain
4	3D	500	100	92.45	69.78	Lowest recorded accuracy
5	3D	1,000	500	93.67	70.12	Minor improvement noted
6	3D	1,000	1,000	94.23	70.01	No significant impact observed

## 4 Conclusions

This study successfully developed and refined a Convolutional Neural Network (CNN) model for predicting phenotypic traits from genotypic data, with a specific focus on schizophrenia (SCZ). Through iterative improvements in data partitioning, model architecture, and regularization techniques, we achieved a notable accuracy of 80 %. This represents a significant step forward in applying deep learning to complex genetic analysis and disease prediction.

A key contribution of this research is the application of CNNs to psychiatric genetics, leveraging their hierarchical structure to detect patterns in genomic data that traditional methods might overlook. The incorporation of dropout layers, learning rate scheduling, and early stopping mechanisms helped optimize model performance by reducing overfitting and enhancing generalization.

Despite these advancements, challenges remain. Overfitting persists to some extent, and the model still exhibits a 20 % prediction error rate. These limitations suggest that while the model provides a strong foundation for genotype-to-phenotype prediction in SCZ, further refinement is essential to improve robustness and potential clinical applications.

In summary, this study adds to the expanding body of research focused on the application of deep learning methodologies within the domain of precision medicine. Ongoing improvements in predictive accuracy and model interpretability are expected to play a critical role in facilitating earlier diagnostic processes and in supporting the development of tailored therapeutic approaches for complex genetic conditions, including schizophrenia.
